# MiR-205-5p inhibition by locked nucleic acids impairs metastatic potential of breast cancer cells

**DOI:** 10.1038/s41419-018-0854-9

**Published:** 2018-07-26

**Authors:** Antonella De Cola, Alessia Lamolinara, Paola Lanuti, Cosmo Rossi, Manuela Iezzi, Marco Marchisio, Matilde Todaro, Vincenzo De Laurenzi

**Affiliations:** 1 Department of Medical, Oral and Biotechnological Sciences, Center of Excellence on Aging and Translational Medicine (CeSi-Met), G. D’Annunzio University, Chieti-Pescara, Italy; 2 Department of Medicine and Aging Science, Center of Excellence on Aging and Translational Medicine (CeSi-Met), G. D’Annunzio University, Chieti-Pescara, Italy; 3 Department of DiBiMIS, University of Palermo, Palermo, Italy

## Abstract

Mir-205 plays an important role in epithelial biogenesis and in mammary gland development but its role in cancer still remains controversial depending on the specific cellular context and target genes. We have previously reported that miR-205-5p is upregulated in breast cancer stem cells targeting ERBB pathway and leading to targeted therapy resistance. Here we show that miR-205-5p regulates tumorigenic properties of breast cancer cells, as well as epithelial to mesenchymal transition. Silencing this miRNA in breast cancer results in reduced tumor growth and metastatic spreading in mouse models. Moreover, we show that miR-205-5p knock-down can be obtained with the use of specific locked nucleic acids oligonucleotides in vivo suggesting a future potential use of this approach in therapy.

## Introduction

Despite advances in breast cancer treatment, metastases still remain the major cause of patients death due to therapeutic failure and disease recurrence. It is currently believed that a subpopulation of cells within the tumor displaying stem-like properties, such as self renewal and ability to differentiate, drives tumor progression and metastasis, and is in general more resistant to therapies, leading to a worst clinical outcome^1–4^. These cells have been indicated as: cancer stem cells (CSCs) or tumor-initiating cells^5^.

Breast cancer was the first solid tumor in which CSCs were identified and characterized by the expression of specific surface markers including CD44+, CD24– and EpCam+^6^. Breast CSCs (BCSCs) are also characterized by epithelial–mesenchymal plasticity^7^ and undergo epithelial to mesenchymal transition (EMT), as well as mesenchymal to epithelial transition (MET) during the different stages of metastatic spreading. EMT is a program involved in tissue morphogenesis induced during embryogenesis by which epithelial cells take on a mesenchymal phenotype, loosing their polarity and acquiring the ability of motility and invasion. Thanks to EMT and MET cancer cells can switch from epithelial to mesenchymal states and vice versa, increasing their ability to invade stromal and vessel tissues, leading to the formation of secondary tumors^8^. In cancer progression, EMT is reactivated and is promoted by the tumor microenvironment leading to the induction of several transcription factors like Zeb1 and Snail and to the activation of intracellular signaling pathways^9^ enabling cells to move to new sites, allowing the formation of metastatic lesions.

Accumulating evidences in the last years support the involvement of microRNAs in EMT modulation by targeting genes related to EMT machinery^10,11^. MiRNAs are small regulatory non-coding RNAs, controlling gene transcription through base pairing with the 3′-untranslated regions (3′-UTR) of specific target mRNAs, leading to the downregulation of gene expression^12^. However, miRNAs activity may change depending on the cellular context and stimuli, indeed the same gene can be regulated by different miRNAs, thus explaining the dualistic and often controversial role of miRNAs.

MiR-205, is one of the most studied miRNAs in breast cancer for its involvement in cellular proliferation, migration and differentiation and it was defined “angel or devil” in cancer, acting either as an onco-suppressor or as an oncogene^13^. Indeed, miR-205 has been shown to inhibit EMT in several tumors, targeting Zeb1 and Zeb2^14^ but has also been reported to promote proliferation and metastasis in other models^15,16^. We have previously reported that patient derived BCSCs express high levels of miR-205-5p compared with more differentiated tumor cells^17^. We found that miR-205-5p upregulation controls CSCs phenotype targeting Her2, p63 and Epidermal growth factor receptor (EGFR) contributing to targeted therapy resistance^17^. Here we further investigate the role of this miRNA in breast cancer progression and show that miR205-5p silencing impairs the metastatic potential of BCSCs in vitro and in vivo modulating EMT program. Moreover, we show that miR205-5p silencing can be obtained with the use of specific locked nucleic acids (LNA) oligonucleotides in vivo suggesting this approach as a future potential therapeutic tool.

## Results

### miR-205-5p regulates the tumorigenic potential of BCSCs

Recently we reported that miR-205-5p is overexpressed in BCSCs compared with more differentiated cancer cells and by regulating the expression of different molecules including p63, participates to sustain a stem-like phenotype of breast cancer cells, desensitizing these cells to targeted therapy^17^.

**Fig. 1:**
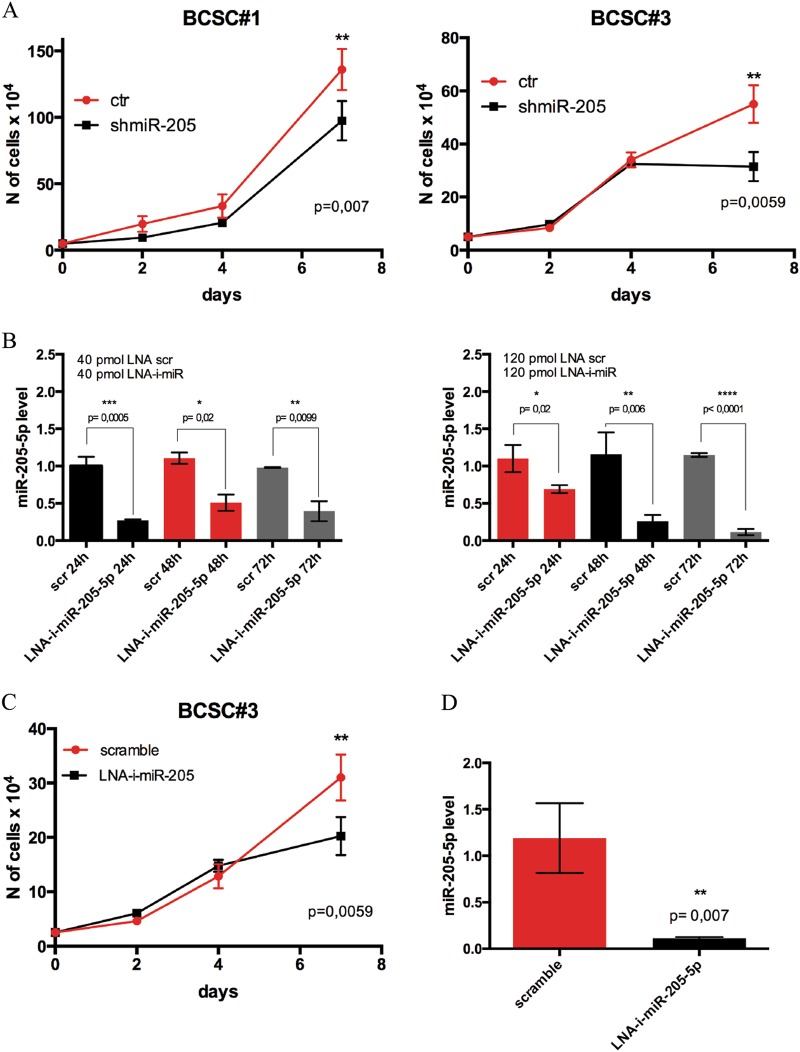
miR-205-5p inhibition impairs BCSCs proliferation in vitro. **a** Cell proliferation assay, by cell count of Trypan blue-stained cells, of BCSC#1 and BCSC#3 infected with mir-205-5p silencing lentivector (shmiR-205) or with the empty vector (ctr). Data are presented as mean ± SD (ANOVA analysis) of different experiments (*n* = 2) each performed in triplicate. **b** qRT-PCR of miR205-5p expression levels of BCSCs#3 treated with 40 pmol (left) or 120 pmol (right) of miRCURY LNA miR-205-5p inhibitor (LNA-i-miR-205-5p) or the scrambled control (scr) for 24, 48 and 72 h. Data are presented as mean ± SD (*T*-test) of three independent experiments each performed in triplicate. **c** Cell proliferation assay (cell count) of BCSCs#3 treated every 72 h with 40 pmol of LNA miR-205-5p inhibitor or a scramble control and cultured up to 7 days. Data are presented as mean ± SD (ANOVA test) of two independent experiments each performed in duplicate. **d** qRT-PCR of miR-205-5p levels of BCSCs treated as described above after 7 days of in vitro culture. Data are presented as mean ± SD (*T*-test) of two independent experiments each performed in triplicate

To further delineate the contribution of miR-205-5p in the regulation of BCSCs properties, we investigated if this miRNA also influenced their tumorigenic potential. To this end, we stably knocked down miR-205-5p with short hairpin RNA (shRNA) lentiviral vectors in two patient-derived BCSC lines and we examined the effect of miR-205 silencing on proliferation in vitro. As shown in Fig. 1a, miR-205 suppression reduced the proliferation of both BCSCs tested.

Then, in order to efficiently silence miR-205 in a way with potential in vivo applications, we took advantage of the LNA-phosphorothioate (PS) oligonucleotides technology. Recently, LNA have been used to inhibit endogenous small non-coding RNAs in vitro and in vivo^18–20^ and therapeutic silencing of disease-associated miRNAs by LNA antisense oligonucleotide is under investigations in clinical trials for human cancers, autoimmune and cardiac disorders^21–23^.

Thus, we tested the effect of miRCURY LNA anti-miR-205-5p (LNA-i-miR-205-5p) oligonucleotides in vitro, treating BCSCs cells with two different molar concentrations (40 and 120 pmol). As shown in Fig. 1b (and Supplementary 1A), LNA-i-miR205-5p reduced miR-205-5p expression levels at both concentrations used and as early as 24 h after treatment reaching the maximum effect of inhibition at 120 pmol of LNA anti-miR-205 after 72 h of treatment.

To confirm the anti-proliferative effect of miR-205 knock-down, we treated BCSCs with 40 pmol of LNA-i-miR-205-5p or a scrambled control every 72 h. Figure 1c (and Supplementary 1B) shows that cell proliferation was significantly reduced after 7 days of treatment. Figure 1d (and Supplementary 1B) confirms that miR-205 levels are downregulated after 7 days of culture in treated samples.

**Fig. 2:**
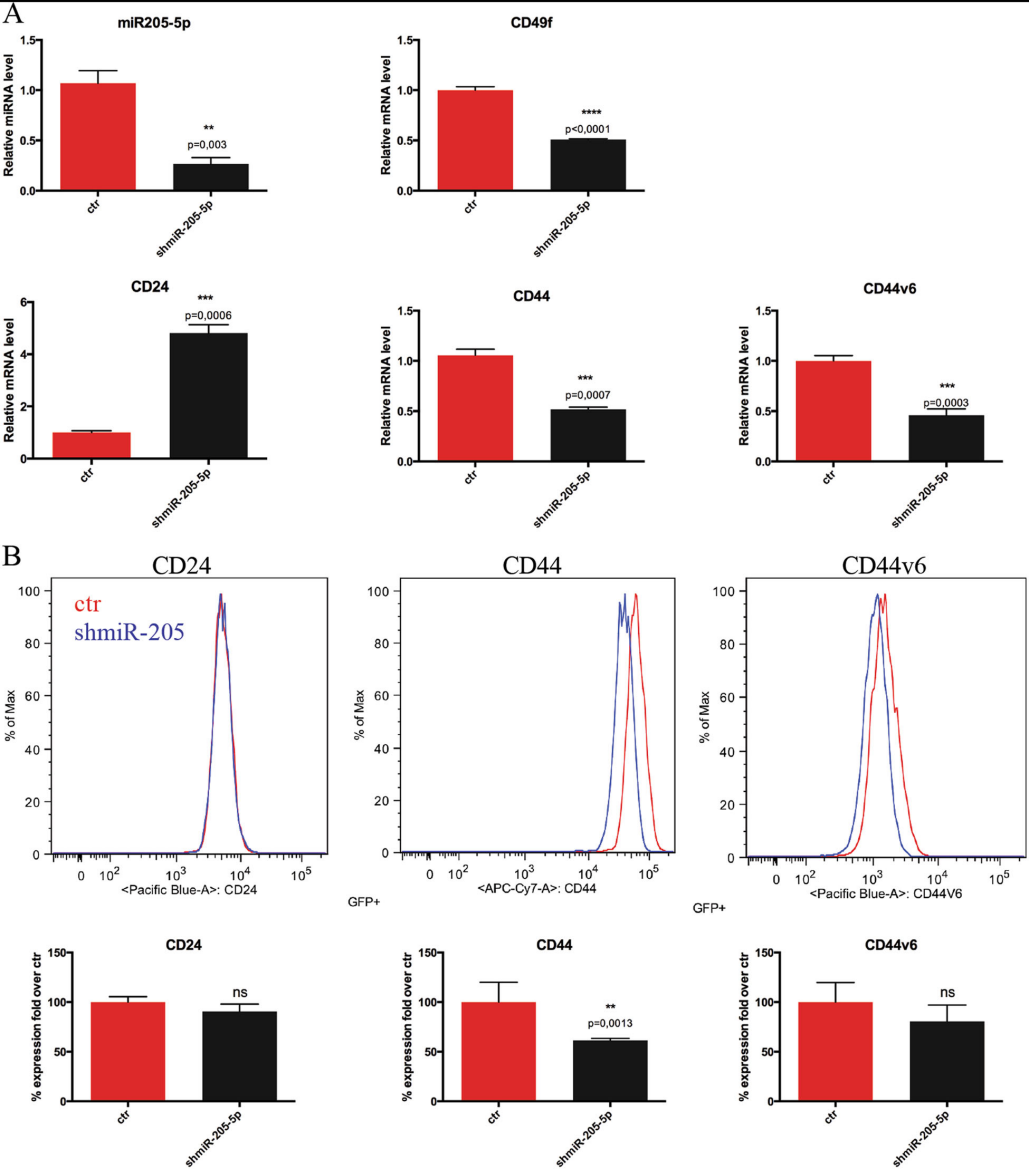
miR-205-5p contributes to control BCSCs surface markers. **a** mRNA expression levels of miR205-5p, CD24, CD44, CD44v6 and CD49f in BCSCs #3 infected with *miR-205-5p* silencing lentivector (shmiR-205-5p) or with the empty vector analyzed by quantitative real-time PCR (qRT-PCR). Data are presented as mean ± SD with *T*-test analysis of three independent experiments. **b** Representative FACS analysis of CD24, CD44 and CD44v6 receptors expression of GFP-positive (infected) BCSC#3 cells infected with *miR-205-5p* silencing lentivector (shmiR: blue line) or with the empty vector (ctr: red line) (upper panel). Histograms (lower panel) show the mean ± SD with *T*-test analysis of the percentage fold over control of receptors expression in two different experiments

**Fig. 3:**
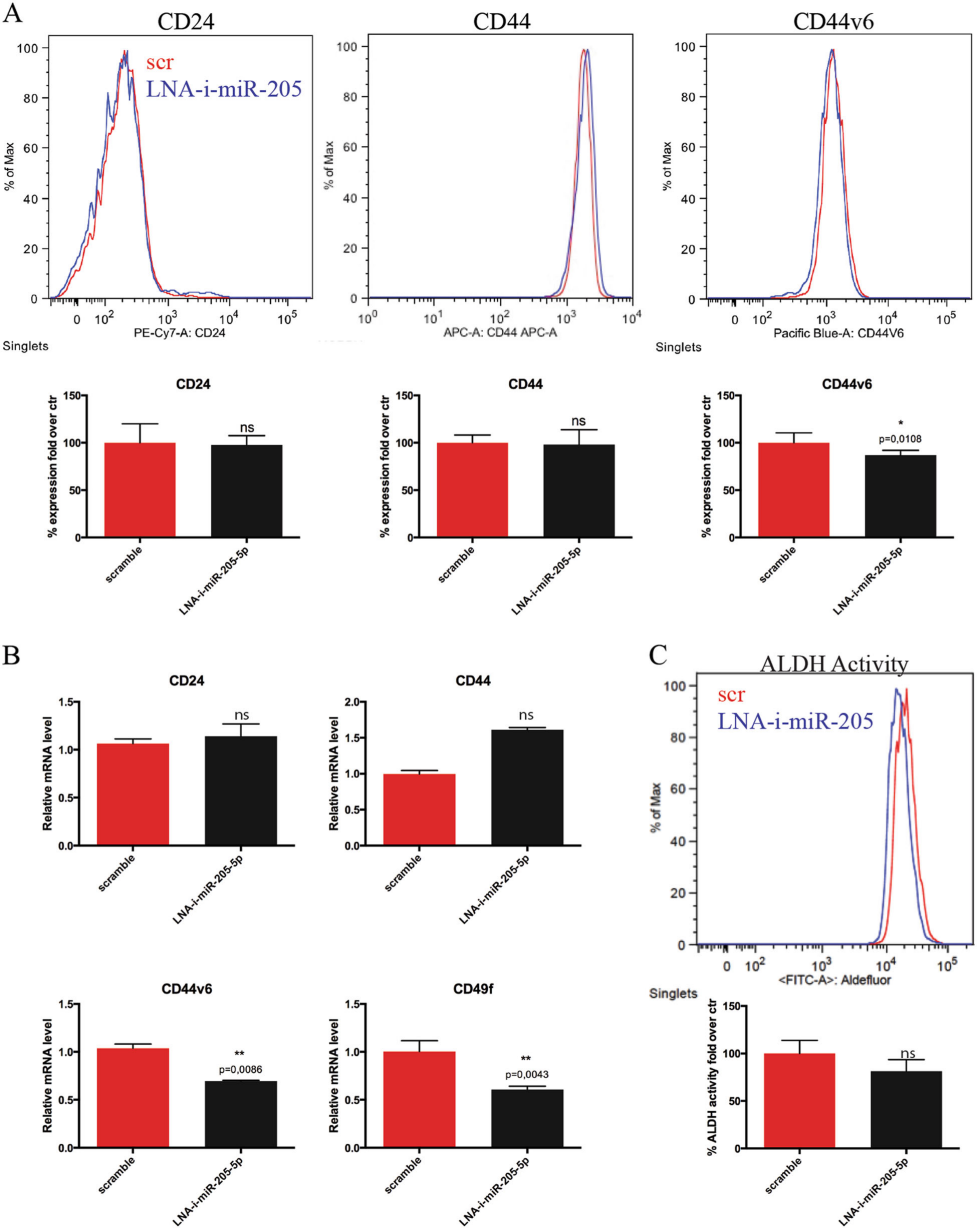
miR-205-5p LNA inhibition reduces the stem-like phenotype of BCSCs. **a** FACS analysis of CD24, CD44 and CD44v6 expression of BCSCs#3 after 24 h of treatment with 120 pmol of miRCURY LNA miR-205-5p inhibitor (LNA-i-miR-205-5p: blue line) or the scramble control (scr: red line) (upper panel). Data are presented as mean ± SD with *T*-test analysis of the percentage fold over control of receptors expression in three different experiments (lower panel). **b** Real-time PCR of CD24, CD44, CD44v6 and CD49f receptors of BCSCs#3 treated for 24 h as described above. Data are presented as mean ± SD with *T*-test analysis. **c** Representative FACS analysis of ALDH1 activity of BCSCs after 24 h of treatment with 120 pmol of LNA miR-205-5p inhibitor or the scramble control using Aldefluor Assay. Histograms (lower panel) show the mean ± SD with *T*-test analysis of the percentage fold over control of ALDH activity in two different experiments

The effect on proliferation was paralleled by altered expression of typical BCSCs surface markers such as CD44 and its splice variant CD44v6^24^, CD24 and alpha-6-Integrin (CD49f) evaluated both by quantitative reverse transcriptase-PCR (qRT-PCR) and fluorescence-activated cell sorting (FACS) silencing mir-205-5p by shRNA or LNA. Silencing miR-205-5p by shRNA results in significant increase in CD24 levels and decrease of CD49f, full-length CD44 levels and CD44v6 splice variant, the last known as a bona fide marker of BCSCs (Figs. 2a, b and Supplementary 2A-B). Similar results were obtained, treating BCSCs with LNA-i-mir-205-5p or a scramble control. FACS analysis in Fig. 3a and qRT-PCR in Fig. 3b show that LNA-i-miR-205-5p treatment reduced significantly the expression of CD44v6 and CD49f both at mRNA and protein level, whereas CD24 and CD44 expression levels remained unchanged. Moreover, treatment with LNA-i-miR-205-5 results in a 20% reduction of aldehyde dehydrogenase (ALDH1) activity confirming reduced stemness (Fig. 3c). Altogether these data, suggest that reducing mir-205-5p expression, switch CSCs into a less “aggressive” phenotype.

**Fig. 4:**
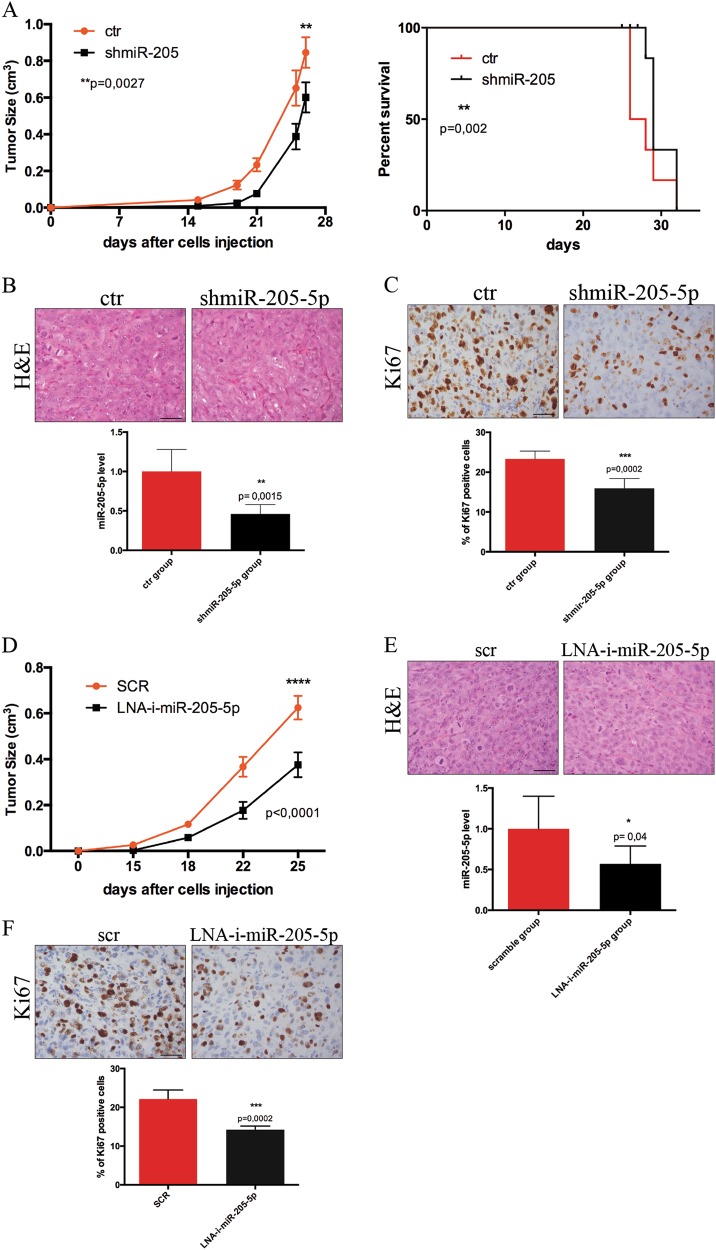
miR-205-5p inhibition compromises tumor growth in vivo. **a** Tumor volume (left panel) of NSG mice (six mice per group) after subcutaneous injection of 1 × 10^5^ BCSC#3 control and BCSC#3 infected with *miR-205-5p* silencing lentivector. Data represent the mean ± SEM (ANOVA test). Survival curve obtained by sacrificing mice when tumors reached the cut off value of 1 cm^3^. The survival curves were analyzed by Mantel–Cox test. **b** Representative H&E staining (upper) and qRT-PCR of miR-205-5p levels (lower) of tumor xenograft samples of control mice group (ctr) and of miR-205-5p silenced group (shmiR-205-5p). Data are presented as mean ± SD with *T*-test analysis. **c** Representative immunohistochemical staining of Ki67 expression (upper) of tumors xenograft sections of control and shmiR-205-5p groups; (scale bar: 50 μm); data are presented as percentage of Ki67-positive cells analyzed with *T*-test (lower). **d** Tumor growth of NSG mice (five per group) after subcutaneous injection of 1 × 10^5^ BCSC#3 and treated with 20 mg/kg LNA anti-miR205-5p or a scramble control twice a week for 3 weeks. Mice were monitored up to 25 days and then sacrificed. Data are shown as mean ± SEM (ANOVA test). **e** Representative H&E of tumors xenograft from scramble and LNA anti-mir-205-5p mice groups and expression levels of miR-205-5p of tumor xenograft lysates analyzed by qRT-PCR. Data are presented as mean ± SD with *T*-test analysis. **f** Ki67 staining of tumors xenograft sections of groups treated as described above. Data are presented as percentage of Ki67-positive cells analyzed with *T*-test (scale bar: 50 μm)

We then tested the effect of miR-205-5p silencing in vivo, by injecting these cells subcutaneously into NOD-SCID-GAMMA (NSG) immune-compromised mice. As shown in Fig. 4a, miR-205-5p silencing resulted in a modest but significant tumor growth rate reduction, as well as an increased survival evaluated by sacrificing the mice when tumors reached a cut off of 1 cm^3^. Figure 4b confirms downregulation of miR-205-5p in tumors while staining of sections for Ki67 shows a lower proliferation activity in tumors from silenced cells compared with controls (Fig. 4c).

We then tested the in vivo effect of systemic delivery of miR-205-5p LNA inhibitor. We subcutaneously injected NSG mice with BCSCs and the day after we intraperitoneally treated mice with six doses (20 mg/kg) of LNA anti-miR-205 or with a scrambled control. Treatment was performed immediately after cells injection as we envisage the potential use of this LNA in adjuvant setting. Significant inhibition of tumor growth was observed after the last LNA injection (on the 18th experimental day) and tumor volumes differed significantly between control and treated groups up to 25 days (Fig. 4d), when mice were sacrificed. Figure 4e shows that tumors xenograft of mice treated with miR-205 LNA inhibitor expressed about 50% less of miR-205-5p and immunohistochemistry analysis (Fig. 4f) confirmed that tumor xenograft of LNA-i-mir-205-5p group showed significant reduction of Ki67-positive cells, thus a reduced proliferation rate. Altogether these results suggest an involvement of miR-205 in tumor formation and establishment.

### miR-205-5p controls BCSCs metastatic potential promoting EMT

**Fig. 5:**
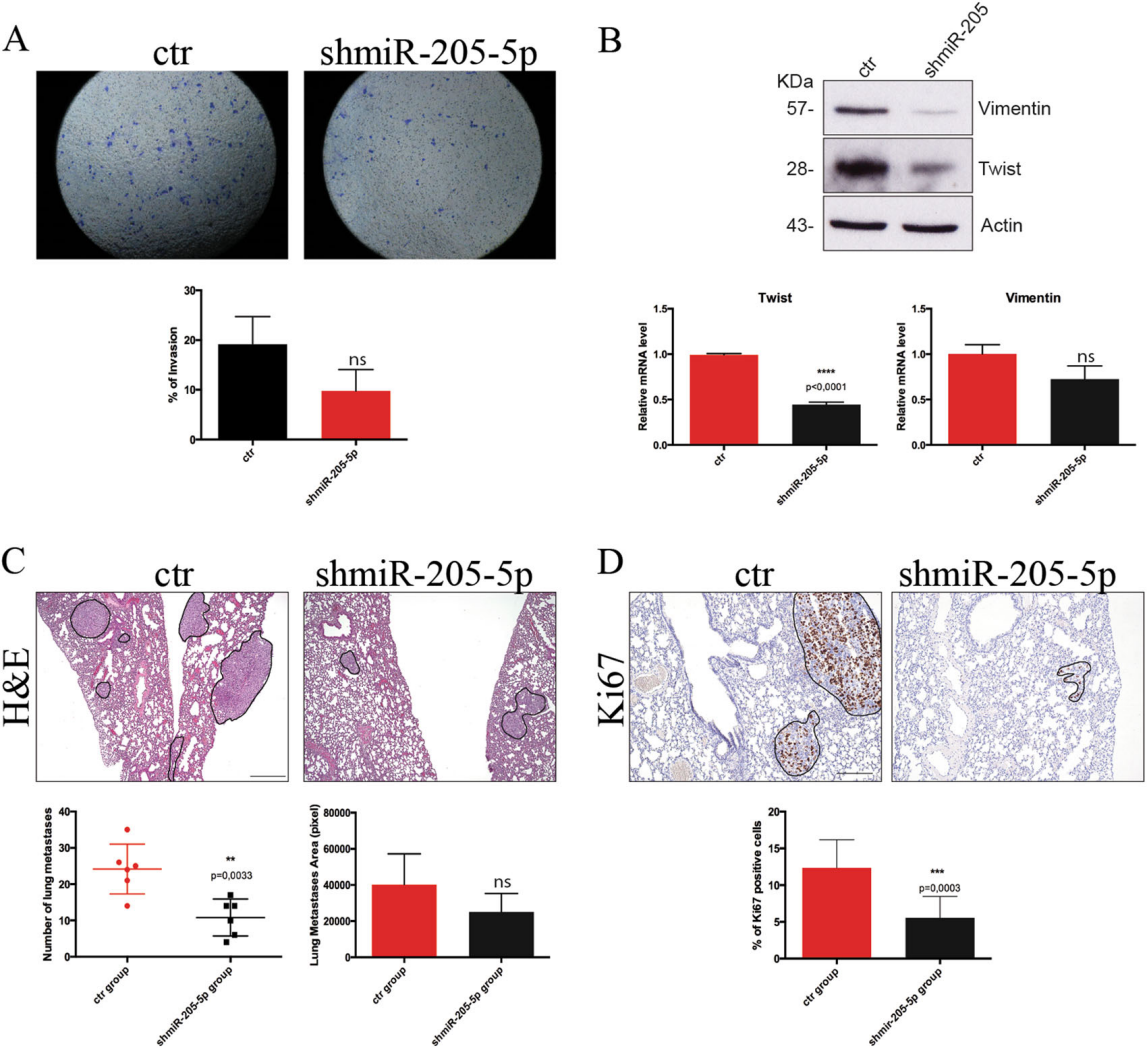
miR-205-5p regulates invasion ability of BCSCs in vitro and in vivo. **a** Representative images (upper) of transwell invasion assay (24 h) of BCSCs#3 infected with *miR-205-5p* silencing lentivector (shmiR-205-5p) or with the control vector (ctr) stained with crystal violet. Quantitative data are presented as mean ± SD of percentage of invasion of three independent experiments (lower). **b** Western blot analysis of Vimentin and Twist protein levels of BCSCs#3 infected with *miR-205-5p* silencing lentivector or with the control vector. Actin was used as a loading control (upper panel). qRT-PCR of Twist and Vimentin mRNA expression levels of BCSCs#3 infected as described above. Data presented as mean ± SD (*T*-test analysis) of three independent experiments (lower panel). **c** Representative H&E staining (upper) of lung metastases of NSG mice (6 per group) after tail vein injection of 2 × 10^5^ BCSCs #3 infected with *miR-205-5p* silencing lentivector or with the control vector (scale bar: 400 μm). Mice were monitored up to 35 days and then sacrificed. Graphs show, respectively, the number (bottom left) and the size (bottom right) of lung metastases for each group. Data were analyzed with *T*-test. **d** Representative immunohistochemical staining of Ki67 in lung metastases of NSG mice treated as described above (scale bar: 200 μm). Percentage of Ki67-positive cells are shown in the graph (bottom) analyzed with *T*-test

We then investigated if miR-205 also regulated the invasive potential of BCSCs. To this end, we initially performed a transwell invasion assay showing that miR-205 silencing results in a decrease (almost 50%) of the invasive ability of BCSCs (Fig. 5a). As shown in Fig. 5b this is also accompanied by reduced expression of EMT related genes, such as Twist and Vimentin, both at protein and mRNA level, whereas the expression level of Slug and Snail remain unchanged (data not shown) suggesting that silencing this miR mainly affects the EMT pathway driven by Twist family and not by the Snail/Slug one.

To further investigate a role of miR-205 in invasion, we used an experimental metastatic model by injecting 2 × 10^5^ BCSCs infected with shmiR-205 construct or with an empty vector in the tail vein of NSG immune-compromised mice. Injection of cancer cells in this model has been shown to result in the formation of metastatic lesions in the lung of animals^25^.

Indeed, after 32 days mice were sacrificed and lung metastasis were counted. Consistently with the reduction of invasive potential shown in vitro, we observed a significant reduction of the number and the size (Fig. 5c) of lung metastasis. In addition, immunohistochemistry analysis of lung lesions (Fig. 5d) shows that metastatic lesions of the miR-205 silenced group have a significant reduced proliferation rate (less Ki67-positive cells).

**Fig. 6:**
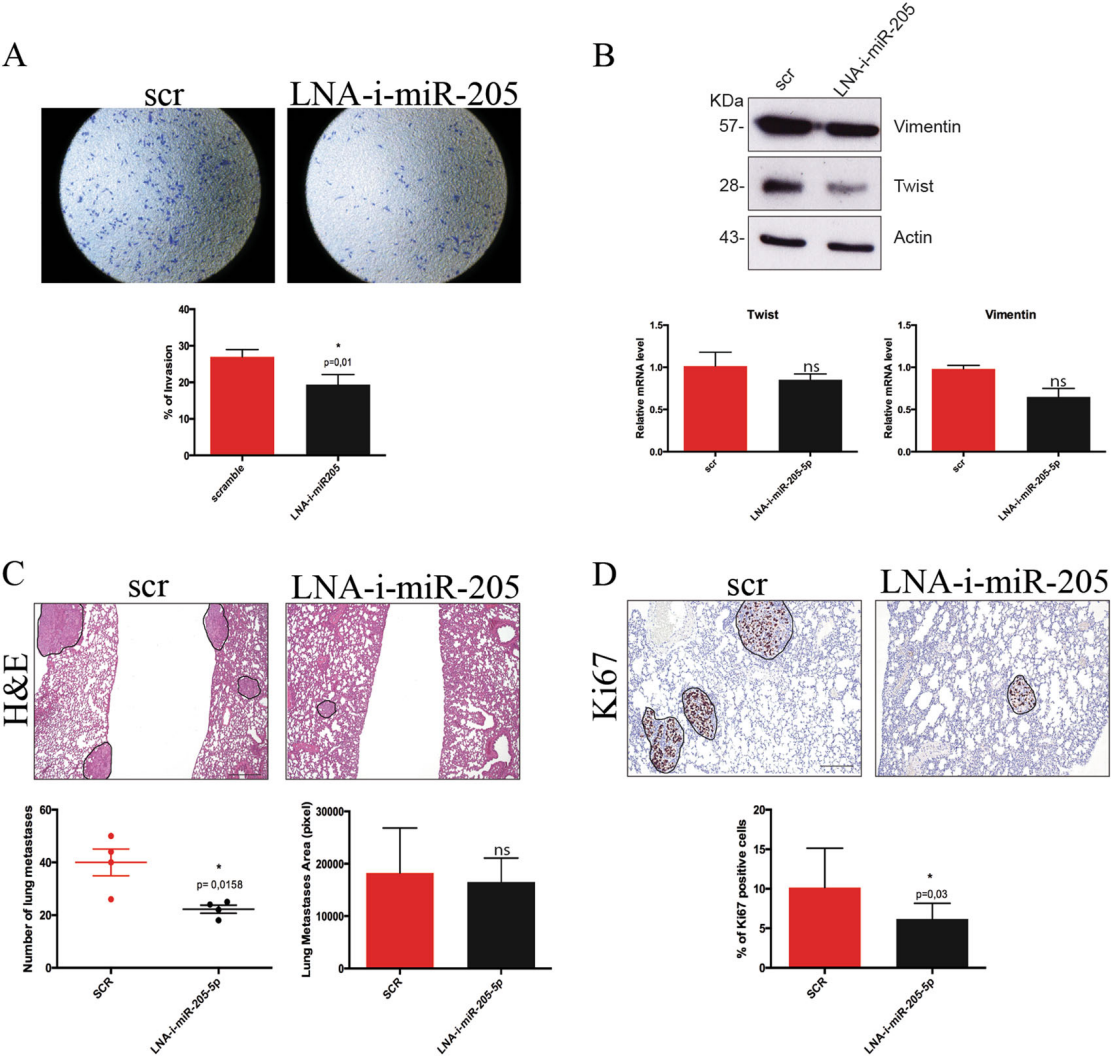
miR-205-5p LNA inhibitor reduces BCSCs metastasis in vitro and in vivo regulating EMT. **a** Representative images of transwell invasion assay of BCSCs#3 after 24-h treatment with 40 pmol LNA anti-miR-205-5p or a scramble control. Quantitative data (bottom) are presented as mean ± SD of percentage of invasion of two independent experiments each conducted in triplicate, analyzed with *T*-test. **b** Western blot analysis of Vimentin and Twist protein levels of BCSCs#3 after 24 h treatment with 40 pmol LNA anti-miR-205-5p or a scramble control; Actin was used as a loading control (upper panel). qRT-PCR of Twist, and Vimentin mRNA expression levels of BCSCs#3 treated as described above (lower panel); data are presented as mean ± SD (*T*-test analysis) of three independent experiments. **c** Representative H&E staining (upper) of lung metastasis of NSG mice (four per group) after tail vein injection with 2 × 10^5^ BCSC#3 and treated with 20 mg/kg LNA-i-miR-205-5p or with LNA scrambled control twice a week for 2 weeks (scale bar: 400 μm). Mice were sacrificed after 30 days. Graphs show the quantification of the number (bottom left) and the size (bottom right) of lung metastasis analyzed with *T*-test. **d** Representative immunohistochemical analysis of Ki67 expression of lung metastases of NSG mice treated as described above (scale bar: 200 μm). Data are presented as percentage of Ki67-positive cells in both mice groups and analyzed with *T*-test (bottom)

Treatment with LNA anti-miR-205-5p also reduced the invasive potential of BCSCs in vitro in both cell lines tested (Fig. 6a and Supplementary 1C). Moreover, we checked the expression level of EMT regulator genes in vitro (Fig. 6b and Supplementary 1D) and ex vivo from tumor xenograft (Figure Supplementary 1E). LNA-i-miR-205-5p treatment leads to a reduction trend of Twist and Vimentin mRNA levels and a significant reduction in their protein levels in line with the results obtained by shmiR-205-5p. Conversely Zeb1, a direct target of miR-205-5p, as previously reported, is upregulated upon miR-205-5p silencing (Supplementary 1F), suggesting as recently described that EMT transcription factors have non-redundant functions and are not all activated at the same time^26-28^.

Finally, we evaluated the effect of LNA-i-miR-205-5p on the metastatic potential of BCSCs in vivo using the same model described above and treating animals with four doses of LNA-i-miR-205 or a scramble control. At the end of the experiment (30 days after cells injection), we collected lungs and counted and analyzed the metastasis. As shown in Fig. 6c, treatment with LNA anti-miR-205-5p significantly reduced the number and the size of lung metastasis. In addition, metastases of LNA-i-miR-205-5p treated group showed lower percentage of Ki67-positive cells (Fig. 6d). All these data confirmed that miR-205 inhibition impairs cell proliferation, motility and invasion ability.

## Discussion

Currently, targeting oncogenic miRNAs represents a new strategy to overcome human cancer and is widely recognized that a subpopulation of cancer cells within the tumor, with stem-like features, plays a pivotal role in cancer progression and recurrence. We have previously shown that miR-205-5p is upregulated in BCSCs compared with more differentiated tumor cells, contributing to sustain a stem-like phenotype, also driving targeted therapy resistance^17^. These findings led us to hypothesize that miR-205-5p could be involved in tumorigenic and metastatic potential of BCSCs.

In this study, we silenced mir-205-5p in vitro and in vivo with either shRNA or LNA PS antisense oligonucleotides, chemically modified to ensure stability, specificity and high binding affinity for the target miRNA. Our in vitro data show that miR-205-5p knock-down results in reduced proliferative ability of these cells paralleled by a reduced expression of CSC markers such as CD44v6, CD44 and alpha-6-Integrin. These results provide additional insights into the role of miR-205 in promoting the expansion of stem cells, as previously described for the progenitors and stem cells populations in skin during early development^29^. Indeed, knocking-out this miRNA in mice causes neonatal lethality with compromised epidermal and hair follicle growth, and impaired proliferation of basal cells in stratified epithelium, by modulating the phosphatidylinositol 3 kinase pathway^29^. Thus, our findings support the hypothesis that in CSCs, genes involved in normal early development like miR-205 are switched on and upregulated.

Along with others, we have previously shown that miR-205 regulates EMT targeting Zeb1^14,17^, here we show that miR-205-5p silencing reduces the invasive ability of BCSCs, modulating the expression of EMT-associated genes like Vimentin and Twist. In line with in vitro results, we show that reduced expression of miR-205 in vivo significantly impairs tumor growth and metastasis formation. Indeed the acute treatment of mice with LNA anti-miR-based therapy, significantly reduces the growth of the primary tumor, as well as the number and the size of lung metastasis derived from BCSCs injection. Furthermore, the systemic delivery of LNA anti-miR205 resulted in an effective mir-205 silencing with no major adverse effect, suggesting the potential safety of this approach.

Several studies^18,30^ have reported the efficiency of LNA anti-miR-based therapies and miRNA-based therapeutics are currently entering clinical trials;^31^ for instance “Miravirsen”, LNA anti-miR122, is the first miRNA-targeted drug to enter phase II clinical trial for its use against HCV infection^21,32^. Thus, the discovery and the development of new miRNAs therapeutics, used alone or in combination with other drugs, against cancer and other disorders, avoiding off-target effects, still remains the great challenge of medicine. Our work supports the idea that LNA anti-miR205 can be possibly used as an adjuvant therapy, to overcome breast cancer progression and metastatic spreading. However, caution should be used in suggesting this approach as miR-205-5p has been shown to act also as a tumor suppressor in other models and further work is required to clearly understand this pathway in order to select patients that might benefit from this approach.

## Materials and methods

### BCSCs isolation and culture

BCSCs were isolated collecting human breast cancer tissues at the Department of Surgical, Oncological and Stomatological Sciences (University of Palermo, Palermo, Italy), in accordance with the ethical standards of the Institutional Committee on Human Experimentation and their molecular subtypes^5^. Breast cancer tissues were digested as previously described^6,33^ and cells were cultured in ultra-low attachment flasks in a selective medium (serum-free) supplemented with 10 ng/mL fibroblast growth factor (βFGF) (Peprotech), 20 ng/mL epidermal growth factor (EGF) (Peprotech) at 37 °C in a 5% (v/v) CO_2_ humidified chamber. In this culture condition, breast cancer cells grow as sphere structures with cancer stem-like properties^34^. We performed all the experiments with BCSCs#1 and BCSCs#3 derived, respectively, from tumors histopathologically classified as follows: BCSC#1 is an invasive ductal carcinoma, grading G2, Estrogen Receptor (ER) 90%, Progesterone Receptor (PR) 60%, Human Epidermal growth Factor Receptor 2 (HER2)/neu 3+ and ki67 > 10%; BCSC#3 is an invasive ductal carcinoma, grading G2, ER 80%, PR 80%, HER2/neu 3+ and ki67 > 10%^17^.

For the authentication of BCSCs was used the short tandem repeat (STR) system (GlobalFiler STR Kit; Applied Biosystems) and the ABIPRISM 3130 genetic analyzer (Applied Biosystems) as previously reported^5^.

### Constructs and LNA microRNA inhibitor

To generate shRNA miR-205 lentivector, we cloned *miR-205-5p* into pSIH-H1-copGFP Vector (System Biosciences, CA, USA) as previously described^17^.

Custom miRCURY LNA microRNA inhibitor in vivo Ready were designed and purchased from Exiqon (Denmark) with the following sequences: i-hsa-miR-205-5p 5′-CCGGTGGAATGAAGG-3′ and Scrambled control 5′-ACGTCTATACGCCCA-3′. The oligonucleotides were High Performance Liquid Chromatography (HPLC) and Na salt exchanged purity level and had full PS backbones. They were custom designed to work in human cells in vitro and in mice xenograft of human breast cancer.

### LNA transfection

miRCURY LNA miR-205-5p inhibitor and the scramble control were used at concentration of 40 pmol (or 120 pmol where indicated) and the mixture was added directly to cells seeded into six-well ultra-low attachment plated. Successful miR205-5p inhibition was evaluated by qRT-PCR.

### Virus generation and infections

Lentiviruses were produced by transient co-transfection of a three-plasmid expression system in the packaging 293T cells, using the calcium phosphate transfection kit (Invitrogen, Life Technologies) as previously described^17^. Then, BCSCs were infected with viral supernatant as previously described^17^ and infection efficiency was assessed by flow cytometry (FACSCanto II Instrument, BD Biosciences) 48 h post-infection evaluating the percentage of Green Fluorescent Protein GFP-positive cells. Data were analyzed with CELLQuest software (BD Biosciences).

### Real-time PCR

For miRNA detection, RNA was extracted from BCSCs and from tumors by using miRNeasy Mini Kit (Qiagen, Germany). Tumors were homogenized by Tissue Ruptor homogenizer (Qiagen, Germany). RNA (50 ng) was used for reverse transcription using TaqMan MicroRNA Reverse Transcription Kit (Applied Biosystem by Life Technologies) with the following stem loop-specific primers: *miR-205-5p* RT: 5′-GTTGGCTCTGGTGCAGGGTCCGAGGTATTCGCACCAGAGCCAACCAGACT-3′ and U44-RT: 5′-GTTGGCTCTGGTGCAGGGTCCGAGGTATTCGCACCAGAGCCAACAGTCAGTT-3′ (Ref).

Real-time PCR was performed by using SsoAdvanced Universal Probe Supermix (Bio-Rad) and Universal Probe Library, Probe #21 (Roche) using the following primers: *miR-205-5p* Fw 5′-GCGGCGGTGTAGTGTTTCCTA-3′ and universal Reverse primer: 5′-GTGCAGGGTCCGAGGT-3′. *miR-205-5p* expression was calculated relative to U44 snoRNA with the following primer: 5′-GCGGCGGCCTGGATGATGATAG-3′. All qRT-PCR were performed with an amplification protocol as follows: one cycle of 95 °C for 30 s and 40 cycles of 95 °C for 15 s and 60 °C for 30 s on CFX96 Touch detection system (Bio-Rad) and relative quantification of miRNA expression was calculated according to the comparative method of 2-^ΔΔCT^.

For detection of other genes, 500 ng of RNA was used for reverse transcription using TaqMan Reverse Transcription Reagents (Applied Bioisystem, Life Technologies) and real-time PCR was performed by using SsoAdvanced Universal SYBR Green Supermix (Bio-Rad) and the reaction was followed by a melting curve protocol according to the specification of the CFX96 Touch instrument (Bio-Rad). Primers used were as follows: for Vimentin R 5′-AGCGAGAGTGGCAGAGGA-3′ and F 5′-GTTTCCCCTAAACCGCTAGG-3′; for Twist F 5′-AGCTACGCCTTCTCGGTCT-3′ and R 5′-CCTTCTCTGGAAACAATGACATC-3′; for CD24 F 5′-TGGATTTGACATTGCATTTGA-3′ and R 5′-TGGGGGTAGATTCTCATTCATC-3′; for alpha-6 integrin (CD49f) F 5′-TTTGAAGATGGGCCTTATGAA-3′ and R 5′-CCCTGAGTCCAAAGAAAAACC-3′; for CD44 F 5′-CGGACACCATGGACAAGTTT-3′ and R 5′-GAAAGCCTTGCAGAGGTCAG-3′; for CD44v6 variant F 5′-AGGAACAGTGGTTTGGCAAC-3′ and R 5′-CGAATGGGAGTCTTCTCTGG-3′.

All genes expression were normalized relative to human β-actin with the following primers: ActF 5′-CAGCTCACCATGGATGATGATATC-3′ and ActR 5′-AAGCCGGCCTTGCACAT-3′^17^. Relative quantification of gene expression was calculated according to the comparative method of 2-^ΔΔCT^.

### Western blotting

Proteins were extracted with a lysis buffer (TRIS-HCl 50 mM pH 8, NaCl 150 mM, Triton X-100 1%, NaF 100 mM, EDTA 1 mM, MgCl_2_ 1 mM, Glycerol 10%) containing a protease inhibitor cocktail (Sigma-Aldrich) and a phosphatase inhibitor cocktail (Roche). Equal amounts of total protein were subjected to sodium dodecyl sulfate–polyacrylamide gel electrophoresis and then transferred to nitrocellulose membranes. The membranes were blocked with 5% nonfat dry milk in phosphate-buffered saline (PBS) with 0,1% Tween 20 and incubated overnight using the following antibodies: anti-β-actin A5441 (Sigma), anti-Vimentin D21H3 (Cell Signaling), anti-Zeb1 D80D3 (Cell Signaling) and anti-Twist 2C1a (Santa Cruz). After washing, membranes were hybridized with horseradish peroxidase-conjugated secondary antibodies (rabbit and mouse, Bio-Rad, CA, USA). Detection was performed with SuperSignal West Dura extended duration substrate kit (Thermo Scientific, USA).

### FACS analysis

For flow cytometric analysis of CD44, CD24 and CD44v6 surface markers, 2 × 10^5^ cells per sample, for all two BCSCs lines tested (BCSC#1 and BCSC#3), were washed in PBS, resuspended in 100 μl of specific antibody diluted in PBS, and incubated for 30 min at + 4 °C. For BCSCs infected with shmiR-205-5p, construct were used: APC-H7 mouse anti-human CD44 (cat. no. 560532; clone G44-26, BD Pharmingen) and BV421 mouse anti-human CD24 (cat. no. 562789; clone ML5, BD Horizon) evaluating CD44 and CD24 staining only for GFP-positive cells. For BCSCs treated for 24 h with 120 pmol LNA, miR-205 inhibitor were used: PE-Cy 7 mouse anti-human CD24 (cat. no. 561646; clone ML5, BD Pharmingen) and APC-mouse anti-human CD44 (cat. no. 559942; clone G44-26, BD Pharmingen). For CD44v6, staining was used: anti-human CD44v6 (cat. no. BBA13; clone 2F10, mouse IgG1, R&D) for 30 min at + 4 °C then incubated with anti IgG1 Vioblue mouse (cat. no. 130-099-084; MACS, Miltenyi Biotec).

ALDH activity was measured following Aldefluor kit manufacturer’s instructions (Stemcell Technologies) upon 24 h of treatment with 120 pmol LNA miR-205 inhibitor or a scramble control.

A FACSCantoII flow cytometer, running with FACSDiVa software (BD Biosciences), was used for sample acquisition and analysis.

All antibodies were titrated under assay conditions, in order to obtain optimal dilutions. Instrument performances and data reproducibility were sustained and checked by using the Cytometer Setup & Tracking Module (BD Biosciences). In order to assess nonspecific fluorescence, fluorescence minus one controls were used. Compensation was calculated using CompBeads (BD Biosciences) and single stained samples.

### Cell proliferation assay

BCSCs infected with shmiR-205-5p or with an empty vector were seeded into 24-well plate at 5 × 10^4^ cells/well. BCSCs treated with miRCURY LNA miR-205-5p inhibitor were seeded into 24-well plate at 2.5 × 10^4^/well. Viable cell count was performed with Trypan blue reagent (Sigma-Aldrich) at the indicated time points. When indicated, cells were treated with 40 pmol LNA scramble or miR-205-5p inhibitor (Exiqon).

### Invasion assay

BCSCs infected with shmiR-205-5p or treated with 40 pmol LNA-miR-205-5p inhibitor were EGF and FGF starved overnight. Then 2000 cells were seeded into the upper chamber of 24-well plate-Transwell 6.5 mm insert, 8 m polycarbonate membrane (Costar, Corning) coated with Matrigel (BD). The receiver chamber were filled with stem cell culture medium supplemented with 10% Fetal Bovine Serum (FBS).

After 24 h, non-invaded cells were removed and crystal violet stain solution was added for 10 min. Percentage of invasion was calculated counting and averaging the number of stained cells in five random fields within each Transwell insert, dividing this number by the area of the microscope viewing field and then multiplying this number by the entire area of the Transwell insert. Then, the total number of cells per insert was normalized by the number of cells seeded.

### Mouse xenograft

NSG mice were purchased from Jackson Laboratory and bred in the animal facility of CeSI-MeT, G. D’Annunzio University of Chieti. Animal care and experimental procedures were approved by the Ethics Committee for Animal Experimentation of the institute according to the Italian law.

Seven weeks old female NSG mice were randomly divided into two groups (six mice per group) and were injected unilaterally with 1 × 10^5^ BCSC#3 control and BCSC#3 sh-mir-205-5p into the fourth mammary fat pad. Cells were suspended in 200 μl of PBS 1:6 Matrigel (BD Biosciences).

Tumor growth was monitored twice a week using calipers and animals were sacrificed when tumors reached ~1 cm^3^ of volume. Tumor volume was calculated as 0.5 × d1^2^ x d2, where d1 and d2 are the smaller and larger diameters, respectively. Tumor xenografts and organs were collected and fixed in 10% neutral-buffered formalin, paraffin embedded, sectioned and stained with hematoxylin and eosin (H&E; BioOptica).

### In vivo experimental metastasis assay

For experimental lung metastasis assay, BCSC#3 control and BCSC#3 sh-mir-205-5p cells were resuspended in PBS and then 2 × 10^5^ cells (in 0.2 mL) were injected via the lateral tail vein of 7 weeks old female NSG mice (six mice per group). Mice were killed 35 days after injection and lungs were harvested and fixed in 10% neutral-buffered formalin.

To optimize the detection of microscopic metastases and ensure systematic uniform and random sampling, lungs were cut transversally, to the trachea, into 2.0 mm thick parallel slabs with a random position of the first cut in first 2 mm of the lung, resulting in 5–8 slabs for lung. The slabs were then embedded cut surface down and sections were stained with hematoxylin and eosin. Slides were independently evaluated by two pathologists.

### LNA in vivo treatment

Seven weeks old female NSG mice were randomly divided into two groups (five mice per group) and were injected unilaterally with 1 × 10^5^ BCSC#3 into the fourth mammary fat pad. The day after cells injection, we treated mice with 20 mg/kg LNA-i-miR-205-5p or with LNA scrambled control (Exiqon) intraperitoneally, twice a week, for 3 weeks. The experiment was conducted for 25 days when mice were sacrificed. Tumors and organs were collected and one part of each organ was preserved for RNA isolation and the other part was fixed in 10% neutral-buffered formalin, paraffin embedded, sectioned and stained with H&E for histology analysis.

For experimental metastasis assay, 7 weeks old female NSG mice were divided into two groups (four mice per group) and were injected with 2 × 10^5^ BCSC#3 via the lateral tail vein. The day after injection, mice were intraperitoneally treated with 20 mg/kg LNA-i-miR-205-5p or with LNA scrambled control (Exiqon) twice a week for 2 weeks. Mice were sacrificed after 30 days and lungs were collected and sectioned as described above (in vivo experimental metastasis assay paragraph).

### Immunohistochemistry

For immunohistochemistry, slides were deparaffinized, serially rehydrated and, after the appropriate antigen retrieval procedure, stained with monoclonal mouse anti-human Ki67 (M7240, Dako), followed by the appropriate secondary antibody. Immunoreactive antigens were detected using streptavidin peroxidase (Thermo Scientific) and the DAB Chromogen System (Dako). After chromogen incubation, slides were counterstained in hematoxylin (BioOptica) and images were acquired by Leica DMRD optical microscope (Leica). The percentage of KI67-positive cells was evaluated on digital images of 4–6 tumors or lungs per group (4–6 × 200 microscopic fields per sample); clear brown nuclei were regarded as positive cells and the percentage of labeling index (number of positive cells/total cells × 100) was calculated for each field, by two pathologists, independently.

### Statistical analysis

For statistical analysis, GraphPad Prism 6 software was used, using *T*-test or ANOVA test with *p* < 0.05.

## Electronic supplementary material

Supplementary Figure 1

Supplementary Figure 2
