# Supplementary material for: MiR-205-5p inhibition by locked nucleic acids impairs metastatic potential of breast cancer cells
Source: Cell Death Dis. 2018 Jul 26;9(8):821. doi: 10.1038/s41419-018-0854-9 (PMC6062508; doi:10.1038/s41419-018-0854-9)
Supplement: Supplementary file 1 — Supplementary Figure 1 [file 41419_2018_854_MOESM1_ESM.pdf]

Supplementary 1

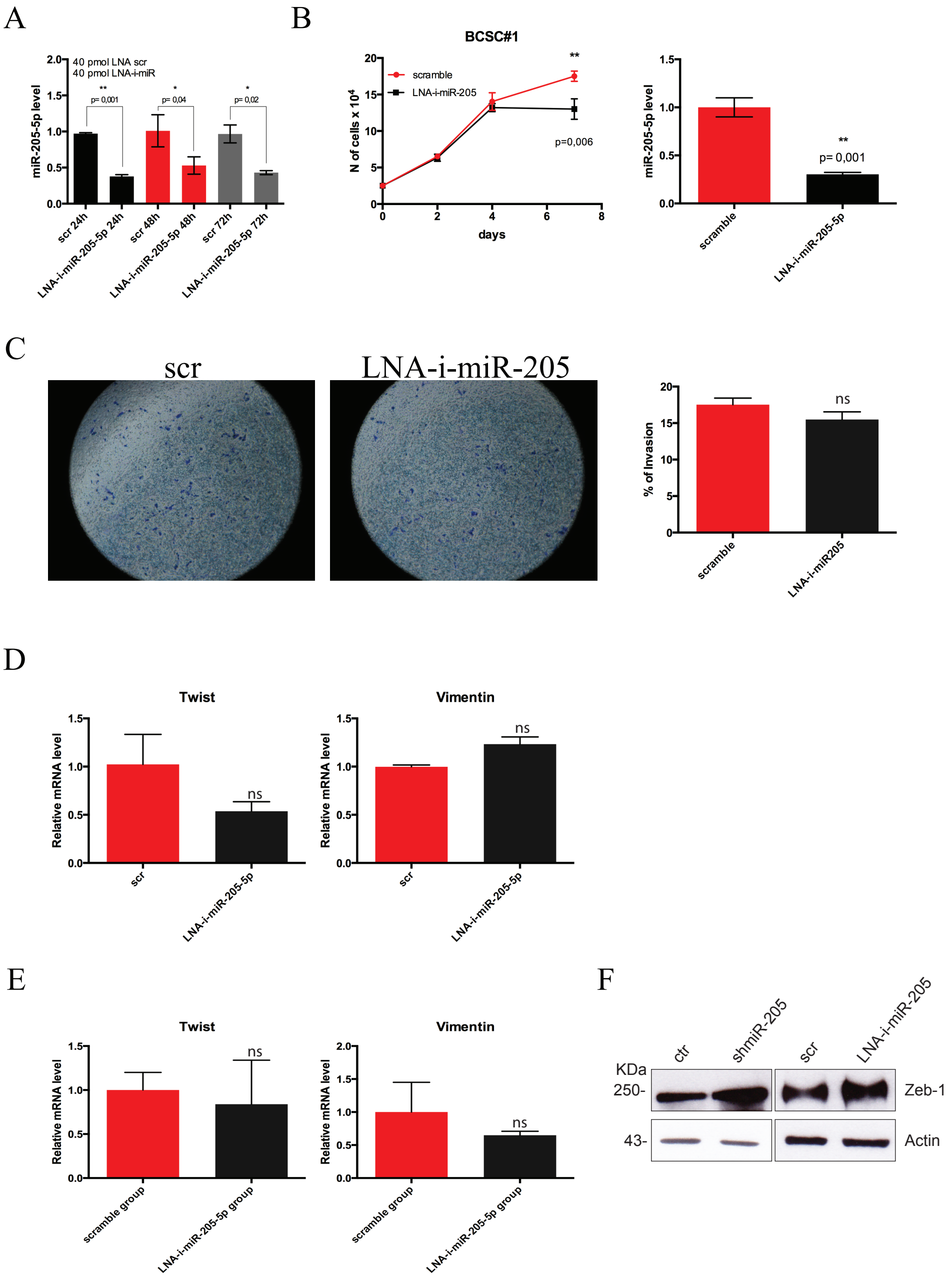

Supplementary 1

A qRT-PCR of miR205-5p expression level of BCSCs#1 treated with 40 pmol of miRCURY LNA miR-205-5p inhibitor (LNA-i-miR-205-5p) or the scramble control for 24, 48 and 72 hours. Data presented as mean ± SD with T test analysis of 3 indipendent experiments performed in triplicate.

B Cell proliferation assay (left) of BCSCs#1 treated every 72 hours with 40 pmol of LNA miR-205-5p inhibitor or a scramble control and cultured up to 7 days. Data presented as mean ± SD (Anova test) of 2 indipendent experiments each performed in duplicate. qRT-PCR (right) of miR-205-5p levels of BCSCs#1 treated as described above after 7 days of in vitro culture. **C** Transwell invasion assay of BCSC#1 after 24 hours treatment with 40pmol LNA anti-miR-205-5p or a scrambled control. Quantitative data are presented as mean ± SD of percentage of invasion of 2 independent experiments each conducted in triplicate, analysed with T test. **D** q-RT-PCR of Twist and Vimentin of BCSCs #1 treated for 24 hours with 40 pmol LNA anti-miR-205-5p or a scramble control.
Data are presented as mean ± SD with T test analysis of 3 experiments.

E qRT-PCR of Twist and Vimentin of lysates from tumors xenograft of mice treated with LNA-i-miR-205-5p or a a scramble control. **F** Western blot analysis of Zeb-1 protein levels of BCSCs#3 infected with miR-205-5p silencing lentivector or with the control vector (right) and treated with 40 pmol LNA anti-mir-205 or a scramble control (left). Actin was used as a loading control.
