# Supplementary material for: MiR-205-5p inhibition by locked nucleic acids impairs metastatic potential of breast cancer cells
Source: Cell Death Dis. 2018 Jul 26;9(8):821. doi: 10.1038/s41419-018-0854-9 (PMC6062508; doi:10.1038/s41419-018-0854-9)
Supplement: Supplementary file 2 — Supplementary Figure 2 [file 41419_2018_854_MOESM2_ESM.pdf]

Supplementary 2

A

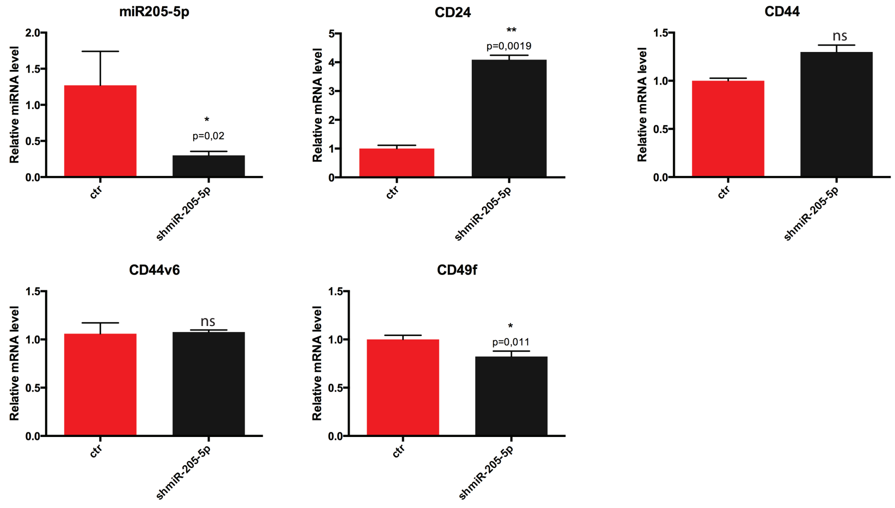

B

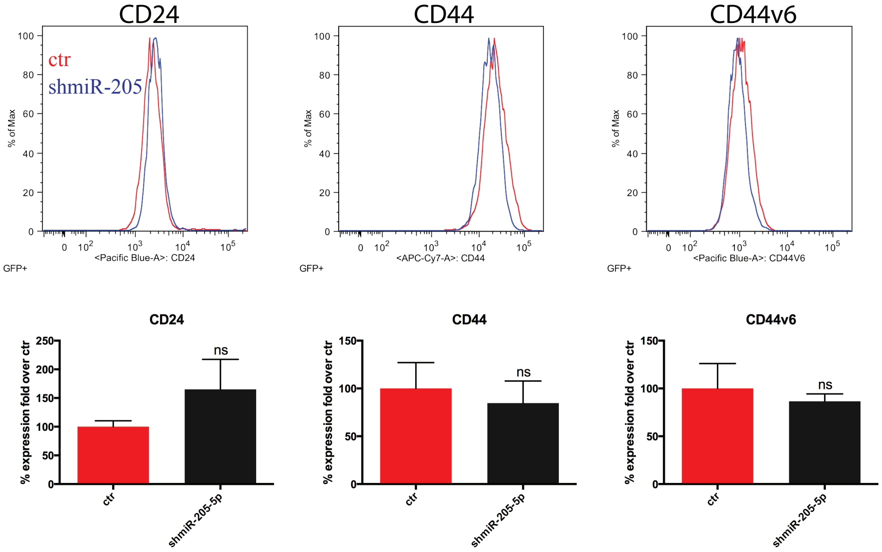

Supplementary 2

A Expression levels of miR205-5p, CD24, CD44, CD44v6 and CD49f in BCSCs #1 infected with miR-205-5p silencing lentivector (shmiR-205-5p) or with an empty vector analysed by quantitative real-time PCR (qRT-PCR).
Data presented as mean ± SD with T test analysis of three independent experiments.

B Representative FACS analysis of CD24, CD44 and CD44v6 receptors expression of GFP positive (infected) BCSC#1 cells infected with miR-205-5p silencing lentivector or with the empty vector (upper panel).
Histograms (lower panel) show the mean ± SD with T test analysis of the percentage of receptors expression of 2 different experiments.
